# Genome sequencing data for wild and cultivated bananas, plantains and abacá

**DOI:** 10.1016/j.dib.2020.106341

**Published:** 2020-09-23

**Authors:** Christine Sambles, Lakshmipriya Venkatesan, Olanrewaju M. Shittu, James Harrison, Karen Moore, Leena Tripathi, Murray Grant, Rachel Warmington, David J. Studholme

**Affiliations:** a Biosciences, University of Exeter, Exeter EX4 4QD, United Kingdom; b International Institute of Tropical Agriculture, P.O. Box 30709, Nairobi, Kenya; c Life Sciences, University of Warwick, Coventry CV4 7AL, United Kingdom; d Eden Project, Bodelva, Cornwall PL24 2SG, UK

**Keywords:** Musa, Genome, Plant, Diversity, Sequence analysis, DNA

## Abstract

We performed shotgun genome sequencing on a total of 19 different *Musa* genotypes including representatives of wild banana species *Musa acuminata* and *M. balibisiana*, allopolyploid bananas and plantains, Fe'i banana, pink banana (also known as hairy banana) and abacá (also known as hemp banana). We aligned sequence reads against a previously sequenced reference genome and assessed ploidy and, in the case of allopolyploids, the contributions of the A and B genomes; this provides important quality-assurance data about the taxonomic identities of the sequenced plant material. These data will be useful for phylogenetics, crop improvement, studies of the complex story of intergenomic recombination in AAB and ABB allotriploid bananas and plantains and can be integrated into resources such as the Banana Genome Hub.

## Specifications Table

**Table:** 

Subject	Biology
Specific subject area	Genomics of crop plants
Type of data	Deoxyribonucleic acid (DNA) sequence
How data were acquired	Shotgun genomic DNA sequencing was performed using Illumina HiSeq 2500, Illumina NovaSeq and BGIseq-500 platforms
Data format	Raw sequencing reads
Parameters for data collection	DNA was extracted from leaf material
Description of data collection	Shotgun genomic DNA sequencing was performed using Illumina HiSeq 2500, Illumina NovaSeq and BGIseq-500 platforms
Data source location	Institution: University of Exeter City: Exeter Country: United Kingdom Latitude and longitude (and GPS coordinates) for collected samples/data: Plant samples were collected from the Eden Project at 50.3601° N, 4.7447° W (50.357165238 -4.740163)
Data accessibility	Repository name: NCBI BioProject Data identification numbers: PRJNA540118, PRJNA413600 Direct URLs to data: https://www.ncbi.nlm.nih.gov/bioproject/540118 https://www.ncbi.nlm.nih.gov/bioproject/413600

## Value of the Data

• This genomic resequencing data will inform studies of *Musa* evolution, biodiversity, speciation and allopolyploidy.
• Genome-wide sequence data are presented for abacá (*Musa textilis*), the Fe'i banana (*M. troglodytarum*) and the pink banana (*M. velutina*) as well as edible and wild bananas and plantains belonging to the species *M. acuminata* and *M. balbisiana* and their interspecific hybrids.
• This is a useful resource for breeders, researchers as well as science communicators engaging with the general public about the germplasm collection at the Eden Project.
• The data can be mined for polymorphisms with value as markers for breeding strategies.
• These data can be integrated into banana genomics resources such as the Banana Genome Hub [1].
• Since some samples were sequenced using more than one method, the data can be used to compare performances of alternative sequencing platforms [2].

## 1 Data Description

Genomic shotgun sequencing data was generated using BGIseq-500 (Table 1), Illumina HiSeq 2500 using libraries of two different sizes (Tables 2 and 3) and Illumina NovaSeq 6000 (Table 4). This generated a total of 505.69 GB and 120.95 GB raw read data for the Eden Project and IITA accessions respectively. Raw data is available at NCBI's Sequence Read Archive [3] via BioProjects PRJNA540118 and PRJNA413600.

**Table 1:** Genomic sequencing data generated using BGIseq-500 (2 × 150 bp reads, 300-bp insert size).

BioSample	SRA accession	Eden project identifier	Received as	Depth of coverage
SAMN11522014	SRR8989628, SRR9734077	2012-1161	*Musa acuminata* ‘Green-Red’	59 ×
SAMN11522015	SRR8989629	2012-1156	*Musa acuminata* ‘Paka’	28 ×
SAMN11522016	SRR8989630, SRR9734074	2012-1173	*Musa acuminata* subsp. *zebrina*	54 ×
SAMN11522017	SRR8989631, SRR9734078	2011-0950	*Musa acuminata*× *balbisiana* ‘Congo 2’ (plantain subgroup)	59 ×
SAMN11522018	SRR8989632	2012-1154	*Musa acuminata* subsp. *malaccensis*	28 ×
SAMN11522019	SRR8989633, SRR9734079, SRR9850640	2001-1027	*Musa balbisiana*	52 ×
SAMN11522020	SRR8989634, SRR9734076, SRR9850639	2012-1164	*Musa acuminata* ‘Calypso’	54 ×
SAMN11522021	SRR8989635	2012-1152	*Musa acuminata*× *balbisiana* ‘Safet Velchi’ (Ney Poovan subgroup)	30 ×
SAMN11522022	SRR8989636	2011-0952	*Musa acuminata*× *balbisiana* “One Hand Planty”	28 ×
SAMN11522023	SRR8989637	1999-2846	*Musa*× *paradisiaca*^a^	31 ×
SAMN11522024	SRR8989638	1998-2307	*Musa acuminata* ‘Pisang Mas’ (Sucrier subgroup)	32 ×
SAMN11522025	SRR8989639, SRR9850642	1999-0524	*Musa textilis*	
SAMN11522026	SRR8989640, SRR9734080, SRR9850641	1999-0158	*Musa troglodytarum* ‘Wain’ (F'ei group)	36 ×
SAMN11522027	SRR8989641, SRR9734075	2012-1166	*Musa velutina*	47 ×

a Accession 1999-2846 was received as *Musa* × *paradisiaca* but genome sequence data suggest that it is *Musa acuminata*.

**Table 2:** Genomic sequencing data generated using Illumina HiSeq (2 × 150 bp reads, 800-bp insert size).

BioSample	SRA accession	Eden project identifier	Received as	Depth of coverage
SAMN11522025	SRR9696635	1999-0524	*Musa textilis*	23 ×
SAMN11522021	SRR9696636	2012-1152	*Musa acuminata*× *balbisiana* ‘Safet Velchi’ (Ney Poovan subgroup)	36 ×

**Table 3:** Genomic sequencing data generated using Illumina HiSeq (2 × 125 bp reads, 300-bp insert).

BioSample	SRA accession	Received as	Depth of coverage
SAMN07758499	SRR6147591	*Musa acuminata*× *balbisiana* ‘Sukali Ndiizi’ (AAB group)	53 ×
SAMN07758501	SRR6147590	*Musa acuminata*× *balbisiana* ‘Gonja Manjaya’ (AAB group)	18 ×
SAMN07758502	SRR6147593	*Musa acuminata* ‘Cavendish’ (AAA group)	23 ×
SAMN07758503	SRR6147592	*Musa balbisiana*	24 ×
SAMN07758500	SRR6147589	*Musa acuminata*× *balbisiana* ‘Pisang Awak’ (ABB group)	28 ×

**Table 4:** Genomic sequencing data generated using Illumina NovaSeq 6000 (2 × 150 bp reads, 300-bp insert size).

BioSample	SRA accession	Eden project identifier	Received as	Depth of coverage
SAMN11522021	SRR9015638	2012-1152	*Musa acuminata*× *balbisiana* ‘Safet Velchi’ (Ney Poovan subgroup)	30 ×
SAMN11522022	SRR9015637	2011-0952	*Musa acuminata*× *balbisiana* ‘One Hand Planty’	28 ×

**Fig. 1:**
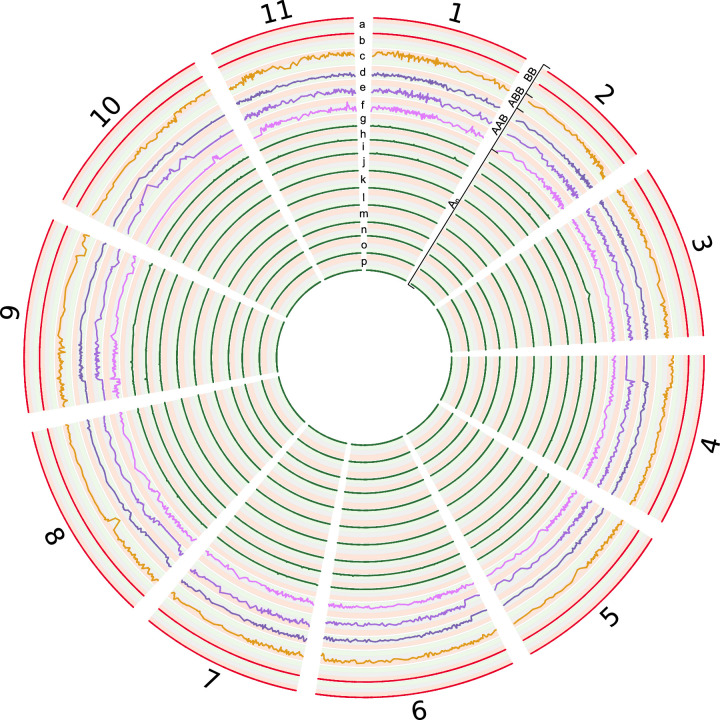
**Circos representation of informative SNP variants identified in the 11 chromosomes of *M. acuminata***. The lines represent the LOESS smoothed percentage of B allele of 16 sequenced *Musa* accessions (*M. acuminata, M. balbisiana* and their hybrids). *Musa* accessions with the highest percentage of A genome are at the centre graduating to those with the highest percentage of B genome on the outside, according to the 1542 identified SNPs. Background colours represent percentage of B allele: green (0–33%), grey (33–66%) and red (66–100%). Tracks from outer (B allele dominant) to inner (A allele dominant) are: a. *M. balbisiana* (SAMN11522019), b. *M. balbisiana* (SAMN07758503), c. *M. acuminata*× *balbisiana* ‘Pisang Awak’ (SAMN07758500), d. *M. acuminata*× *balbisiana* ‘One Hand Planty’ (SAMN11522022), e. *M. acuminata*× *balbisiana* ‘Gonja Manjaya’ – AAB group (SAMN07758501), f. *M. acuminata*× *balbisiana* ‘Sukali Ndiizi’ (SAMN07758499), g. *Musa*× *paradisiaca* (SAMN11522023), h. *M. acuminata*× *balbisiana* ‘Safet Velchi’ – Ney Poovan subgroup (SAMN11522021), i. *M. acuminata* ‘Calypso’ (SAMN11522020), j. *M. acuminata* x *balbisiana* ‘Congo 2’ – plantain subgroup (SAMN11522017), k. *M. acuminata* ‘Pisang Mas’ – Sucrier subgroup (SAMN11522024), l. *M. acuminata* subsp. *malaccensis* (SAMN11522018), m. *M. acuminata* ‘Paka’ (SAMN11522015), n. *M. acuminata* ‘Green-Red’ (SAMN11522014), o. *M. acuminata* subsp. *zebrina* (SAMN11522016), p. *M. acuminata* ‘Cavendish’ – AAA group (SAMN07758502). * A_n_ describes A genome autopolyploidy i.e. AA or AAA or AAAA.

An important quality control step is to check whether the sequence data are consistent with the botanical identifications of the source material. Therefore, we assessed observed against expected levels of ploidy. For allopolyploids purported to originate from interspecific hybrids between *Musa acuminata* and *Musa balbisiana*, we assessed the relative contributions of these respective “A” and “B” genomes compared against the expected characteristics of each sample as described under Experimental Design, Materials, and Methods. The resulting quality-control metrics are summarised in Table 5 and in Fig. 1. Accessions 2012-1152 (SAMN11522021), 1999-2846 (SAMN11522023) and 2011-0950 (SAMN11522017) were expected to be allopolyploids containing contributions from both the A and B genomes but sequence data appeared to be exclusively from the A genome, suggesting that these three plants had been mis-identified. Further, there were discrepancies between the expected ploidy levels versus the empirically inferred levels in several accessions.

**Table 5:** Ploidy prediction and estimated composition of 16 accessions of *Musa* spp.^a^

BioSample	Name	Expected ploidy	Observed ploidy according to nQuire (if different to expected)	Expected composition	SNP data consistent with expected composition?
SAMN11522018	*Musa acuminata* subsp. *malaccensis*	2		AA	Yes
SAMN11522015	*Musa acuminata* ‘Paka’	2		AA	Yes
SAMN11522014	*Musa acuminata* ‘Green-Red’	3		AAA	Yes
SAMN11522016	*Musa acuminata* subsp. *zebrina*	2	4	AA	Yes
SAMN07758502	*Musa acuminata* ‘Cavendish’	3		AAA	Yes
SAMN11522020	*Musa acuminata* ‘Calypso’	4		AAAA	Yes
SAMN11522021	*Musa acuminata*× *balbisiana* ‘Safet Velchi’ (Ney Poovan subgroup)	2	3	AB	No: appears to be exclusively A
SAMN07758499	*Musa acuminata*× *balbisiana* ‘Sukali Ndiizi’	3		AAB	Yes
SAMN07758501	*Musa acuminata*× *balbisiana* ‘Gonja Manjaya’	3		AAB	Yes
SAMN11522022	*Musa acuminata*× *balbisiana* ‘One Hand Planty’	3		AAB	Yes
SAMN07758500	*Musa acuminata*× *balbisiana* ‘Pisang Awak’	3	4	ABB	Yes
SAMN11522019	*Musa balbisiana*	2	4	BB	Yes
SAMN07758503	*Musa balbisiana*	2	4	BB	Yes
SAMN11522024	*Musa acuminata* ‘Pisang Mas’ (Sucrier subgroup)	2		AA	Yes
SAMN11522017	*Musa acuminata*× *balbisiana* ‘Congo 2’ (plaintain subgroup)	3		AAB	No: appears to be exclusively A
SAMN11522023	*Musa*× *paradisiaca*	2	3	AAB or ABB	No: appears to be exclusively A

a Ploidy analysis was only performed on *M. acuminata, M. balbisiana* accessions and their hybrids. Consequently, *Musa textilis* (SAMN11522025), *Musa troglodytarum* ‘Wain’ (F'ei group) (SAMN11522026) and *Musa velutina* (SAMN11522027) were excluded.

## 2 Experimental Design, Materials and Methods

Fresh leaf material was obtained from five accessions from the IITA (International Institute of Tropical Agriculture) [4] accessions and 14 from the Eden Project. DNA was extracted from fresh leaf material and sequenced using a combination of Illumina HiSeq 2500, Illumina NovaSeq 6000 and BGIseq-500 platforms. This yielded at least 20 × coverage of each genome and was sufficient for calling single-nucleotide polymorphisms, detecting presence/absence polymorphisms and cataloguing patterns of heterozygosity.

From the 14 plant accessions from the Eden Project, cigar leaves were cut from the plant and lyophilised in a freeze dryer before sending to BGI Tech Solutions (Hong Kong) Co., Limited, where DNA extraction and sequencing was performed.

For the five accessions from the IITA (International Institute of Tropical Agriculture), genomic DNA was isolated using a modified CTAB (hexadecyltrimethylammonium bromide) extraction method [5]. The University of Exeter's Sequencing Service prepared Illumina sequencing libraries after fragmenting 500 ng of DNA to an average size of 500 bp, using the NEXTflex 8-barcode Rapid DNAseq kit sequencing (Perkin Elmer) with adapters containing indexes and 5–8 cycles polymerase chain reaction (PCR) [6]. Library quality was determined using D1000 screen-tapes (Agilent) and libraries were either sequenced individually or combined in equimolar pools. Sequencing was performed on a single lane of a high-output v4 flow-cell on the Illumina HiSeq 2500 at the University of Exeter, yielding pairs of 125-bp reads.

This yielded at least 20 × coverage of each genome, sufficient for calling single-nucleotide polymorphisms, detecting presence/absence polymorphisms and cataloguing patterns of heterozygosity. Reads were also generated with longer inserts using the Illumina HiSeq (2 × 150 bp reads, 800-bp insert size) for two of the samples, which potentially aids resolution of sequence repeats if data are used in *de novo* assembly of genomes.

The quality of the sequencing reads was evaluated using FASTQC [7]. Before further analyses, reads were trimmed and adapters removed using TrimGalore [8] with command-line options “-q 30 –paired”. Trimmed and filtered reads were aligned to the *M. acuminata* genome [9] using BWA [10] to generate binary alignment map (BAM) files [11].

As a prerequisite for plotting the relative contributions of the A and B genomes in allopolyploids, we first identified a set of informative SNPs that distinguish A (*M. acuminata*) from B (*M. balbisiana*) as follows utilising SAMtools’ *mpileup* function, BCFtools [11,12] and custom scripts available at https://github.com/davidjstudholme/SNPsFromPileups. First, the relevant BAM alignment files were converted into uncompressed VCF format using SAMtools v1.6 (*mpileup* function), selecting for variant sites only (-v) using the alternative model for multiallelic and rare-variant calling (-m). Potential SNPs were filtered using the filter function of BCFtools (v1.6), excluding potential SNPs that were within 100 base pairs of an indel (–SnpGap 100) and had a quality score of less than 35 (QUAL>=35) with a depth of 5 or more reads (MIN(DP)>=5). The minimum number of reads supporting an indel was set to two (MIN(IDV)>=2). Variants that were flagged as indels were excluded (INDEL=0). The resulting filtered VCF files contained the positions of candidate SNPs that distinguished the B genome [13] versus the A reference genome [14]. At each of these informative SNPs, we quantified the relative abundance of the A- and B- alleles, only considering sites where the depth was between 10 and 50. When plotting, the resulting percentage of the B allele was smoothed in R using the LOESS package [15]. The percentages of the B alleles at each SNP were visualised using Circos [16] (Fig. 1).

We used nQuire [17] to estimate ploidy from the BAM files (of genomic reads aligned agains the *M. acuminata* reference genome). After de-noising to remove noise from mis-mapping due to highly repetitive regions, we assessed ploidy level using the *lrdmodel* command of nQuire to produce delta log-likelihoods of diploidy, triploidy or tetraploidy. The lowest delta log-likelihood was taken to indicate the most likely ploidy level (Table 5). To infer ploidy levels, we used nQuire [17] to predict ploidy using BAM alignment files generated with BWA. The ploidy model yielding lowest value of ΔlogL was chosen as the inferred ploidy. The command lines used were as follows:

nQuire create -b example.bam -o example

for i in *.bin; do echo $i; nQuire denoise $i -o $i\_denoised; done

for i in *_denoised.bin; do echo $i; nQuire lrdmodel -t 8 $i; done

## Declaration of Competing Interest

The authors declare that they have no known competing financial interests or personal relationships which have, or could be perceived to have, influenced the work reported in this article.
